# Minimum dietary diversity among women of reproductive age in urban Burkina Faso

**DOI:** 10.1111/mcn.12897

**Published:** 2019-12-19

**Authors:** Estefania Custodio, Francois Kayikatire, Sonia Fortin, Anne‐Claire Thomas, Yves Kameli, Tharcisse Nkunzimana, Biram Ndiaye, Yves Martin‐Prevel

**Affiliations:** ^1^ European Commission Joint Research Centre Ispra Italy; ^2^ NUTRIPASS, Univ Montpellier, Institut de Recherche pour le Développement Montpellier SupAgro Montpellier France; ^3^ UNICEF South Sudan South Sudan

**Keywords:** adolescent girls, Burkina Faso, dietary diversity, micronutrients, urban, women of reproductive age

## Abstract

Micronutrient malnutrition is a challenge for women of reproductive age, who are particularly vulnerable due to greater micronutrient needs. The minimum dietary diversity for women (MDD‐W) indicator is a micronutrient adequacy's proxy for those women, but little is known about its relation to other dimensions. We assessed MDD‐W and its association with other socioeconomic, food security and purchasing practices in urban Burkina Faso. We conducted multi‐stage cluster sampling in two main cities of Burkina Faso, stratified by type of district, and interviewed 12 754 women in the 2009‐2011 period. We obtained food consumption data through unquantified 24 hour recalls and computed MDD‐W as consuming at least five out of ten predefined food groups. We constructed multivariable regression models with sociodemographic and food security covariates. MDD‐W in urban Burkina Faso was 31%, higher in Ouagadougou (33%) than in Bobo‐Dioulasso (29%), and lower in unstructured districts. The most frequently consumed food groups were ‘all starchy', ‘vitamin A rich dark green leafy vegetables' and ‘other vegetables'. Household's expenses were associated with higher likelihood of MDD‐W, while the association with household food security indicators varied by year and type of district. Purchasing foods in markets and choosing the place of purchase based on large choice rather than proximity showed a positive association with the MDD‐W. Only one in three women in urban Burkina Faso reached the minimum dietary diversity, and although socioeconomic and food security variables had the greatest effect on MDD‐W, purchasing practices, like going to the market, also showed a positive effect.

**Key Messages**

- Only one in three women in urban Burkina Faso were reaching the minimum dietary diversity measured by the MDD‐W indicator
- The MDD‐W was significantly lower among women living in unstructured districts as compared with structured ones
- Household's expenses were consistently associated with higher likelihood of MDD‐W, while the association with household food security indicators varied by year and type of district
- Behavioural proxies also showed a positive association with the MDD‐W suggesting that the purchase in markets to be exposed to larger diversity should be encouraged as a means to increase the diversity of women's diets

## 1 INTRODUCTION

Maternal micronutrient deficiencies constitute a widespread nutrition challenge faced by women living in resource‐poor settings, the consequences of which affect not only the health and survival of women but also their children, notably through intrauterine growth retardation (Allen, 2005) (Bartley, Underwood and Deckelbaum, 2005).

One of the main factors of this type of malnutrition is the poor quality of women's diets, notably their lack of diversity. There is ample evidence from developed countries that dietary diversity is indeed strongly associated with nutrient adequacy (Ruel M, n.d.) and a growing evidence from developing countries also supports this association (Mirmiran, Azadbakht, & Azizi, 2006) (Arimond et al., 2010) (Mirmiran, Azadbakht, Esmaillzadeh, & Azizi, 2004).

In resource‐poor environments across the globe, low quality monotonous diets are the norm. When grain or tuber‐based staple foods dominate and diets lack vegetables, fruits and animal‐source foods, the risk for a range of micronutrient deficiencies is high. Women of reproductive age (15‐49 years old) are particularly vulnerable because of their higher micronutrient needs (Torheim, Ferguson, Penrose, & Arimond, 2010).

Despite many calls for attention to women's diet quality in the past decades, with a specific focus on micronutrient adequacy, there has been little programmatic action so far. Among other reasons for that were, the scarcity of data on women dietary patterns and micronutrient deficiencies, and the lack of valid indicators to assess situations in low‐income countries. In 2014 the Minimum Dietary Diversity for Women indicator (MDD‐W) was endorsed (Food and Agricultural Organization (FAO), 2014) and validated as a simple proxy indicator reflecting the micronutrient adequacy in the diet of women of reproductive age (Women's Dietary Diversity Project (WDDP) Study Group, 2017). In addition, a manual was released to help practitioners collect data in a standardized manner defining MDD‐W as ‘a dichotomous indicator of whether or not women 15‐49 years of age have consumed at least five out of ten defined food groups the previous day or night. The proportion of women 15‐49 years of age who reach this minimum in a population can be used as a proxy indicator for higher micronutrient adequacy, one important dimension of diet quality' (FAO, 2016). It has thus the potential to be used in both national and subnational assessments for target setting, impact evaluation and advocacy.

However, due to its relative short existence there is little data on the estimates of MDD‐W in different contexts, and on how the indicator relates to other socioeconomic and food security dimensions, which could inform policy makers on the relevant sectors to be targeted for achieving better women's dietary diversity.

In addition, while there is often a focus on food and nutrition security in rural areas, where the situation is usually more difficult, the rapid urbanization along with the global economic crisis has put many poor urban people at high risk of bad quality diets and malnutrition in developing countries (Global Panel on Agriculture and Food Systems for Nutrition, 2017). Therefore, we conducted the present study to assess the prevalence of MDD‐W and how it relates to other socioeconomic, food security and purchasing practices in women of reproductive age in the two main cities of Burkina Faso.

## 2 MATERIALS AND METHODS

### 2.1 Study area and population

The data sets come from yearly cross‐sectional surveys on Food and Nutrition Vulnerability on Urban Environment conducted in the cities of Ouagadougou and Bobo‐Dioulasso in the years 2009, 2010 and 2011 by the Institut de Recherche pour le Développement. The project has been detailed elsewhere (Kameli et al., 2012) (Becquey. & Martin‐Prével, 2008). Ouagadougou is the capital and largest city of Burkina Faso and Bobo‐Dioulasso the second largest with, populations around 1.500.000 and 500.000 inhabitants at the time of surveys, respectively (Conseil national de la Statistique, 2018). Also, Ouagadougou

### 2.2 Sampling and study design

Samples were obtained by multi‐stage cluster sampling performed separately in each city and at each year of the study.

The city of Ouagadougou was divided in 63 Enumeration Areas (EA) thanks to preliminary work done by Institut de Recherche pour le Développement's geographers, who had divided the whole city into 63 ‘homogeneous areas', particularly in terms of population density. In each area a number of Global Positioning System (GPS) points were randomly selected proportionally to the size of EA in terms of numbers of households. Then one household was selected by random walk for each GPS point. Surveys were repeated in July each year among new, randomly selected households using the same geographical division in 63 homogenous areas.

In Bobo‐Dioulasso, households were also randomly selected using a two‐stage sampling method but as there was no sampling frame available similar to the one used in Ouagadougou, a more classical method was used: first, 60 among the 434 EA covering the whole city of Bobo‐Dioulasso available thanks to the latest population census were randomly selected proportionally to their size in terms of numbers of households. In each EA, 5 starting points were determined using a randomly numbered grid placed on a map of the area, then 10 households were selected around each starting point by random walk technique. Surveys took place in December 2009, November 2010 and October 2011, among new, randomly selected households using the same procedure.

### 2.3 Data collection

We collected information on the household's socio‐demographic and economic characteristics, as well as on food insecurity and dietary diversity indicators, among others.

The sections of the questionnaire referring to household assets and food security were addressed to the head of the household. For the construction of the MDD‐W an unquantified 24‐h dietary recall was conducted in each household. The respondent of this recall was preferably the mother of a preschool‐aged child, a mother, or a woman of the household; if none of these were available the head of household was interviewed. For the present study, only female respondents to the 24‐h dietary recall were taken into account. The respondent was asked to list all the foods and drinks she had consumed the previous day and night and, with the help of the enumerator, to identify all eating or drinking occasions. Based on this open recall, the enumerator checked which food groups were consumed using a predefined list of 20 food groups. Each food group was counted as one if consumed, independently of the frequency of consumption. A final prompt was offered concerning food groups that had not been cited.

### 2.4 Variables construction

As recommended for the calculation of the MDD‐W (FAO, 2016), the 20 food groups of the 24‐h recall were then aggregated in the 10 predefined food groups (1‐Grains, white roots and tubers, and plantains, 2‐Pulses, 3‐Nuts and seeds, 4‐Dairy, 5‐Meat, poultry and fish, 6‐Eggs, 7‐Dark green leafy vegetables (DGLV), 8‐Other vitamin A‐rich fruits and vegetables, 9‐Other vegetables, 10‐Other fruits to compute the dietary diversity score (WDDS‐10). We constructed the dichotomous MDD‐W variable by computing 1 if the women had a WDDS‐10 equal to or higher than 5, and a 0 if her WDDS‐10 was below 5. See the MDDW Guide to measurement for more details (FAO, 2016).

For each city, the socioeconomic level was assessed by an index constructed by correspondence analysis of the following variables: household assets, quality of housing and equipment, access to water and number of people per room (Harttgen & Klasen, 2010). The index was computed using the 2009 sample and households surveyed in 2010 and 2011 were considered as supplementary statistic units in the statistical procedure. The coordinate on the first axis of the correspondence analysis was interpreted as a summary indicator of the socioeconomic level. This means that coefficients for all variables used were calculated on the basis of the 2009 sample, then the same coefficients were applied for the respective variables in household of the 2010 and 2011 samples. Coordinates of 2009 households were used to determine cut‐off of three tertiles of economic status: ‘low', ‘middle' and ‘high'. The youth ratio was calculated as the number of household members under 15 years of age divided by the number of persons of 15 years of age or above, and the dependency ratio as the number of people not contributing to household's income divided by the number of people contributing. Both ratios were further categorized into tertiles.

Total expenses were calculated by summing up the regular expenses on food, transport, housing, energy, water, clothing, hygiene, education, health and leisure. Exceptional expenses such as celebrations or mortgages were not included in the calculation of the total expenses variable used in this analysis.

In order to measure the food security dimension we used the Household Food Insecurity Access Scale (HFIAS) which we constructed following standard guidelines (Coates, Swindale & Bilinsky, 2007). The HFIAS is a brief survey instrument to measure the severity of the self‐reported household http://medanth.wikispaces.com/Food+Insecurity during the past 30 days. It consists of nine occurrence questions and nine frequency questions that ask about changes that households have made in their diet or food consumption patterns as a result of limited resources to acquire food. If an occurrence question is positively answered, the frequency question scores are 1 for rarely (once or twice in the past four weeks), 2 for sometimes (three to ten times in the past four weeks) and 3 for often (more than ten times in the past month). In case of non‐occurrence, the score is 0. The points are then added in a score ranging from 0 to 27 which can be categorized in four categories: food secure, moderately food insecure, mildly food insecure, severely food insecure. For the purpose of this study, these four categories were further combined in a dichotomous variable equalling 1 if severely food insecure and 0 otherwise.

The variables related to monthly food purchases (meat, fish and prepared dishes) were computed as 1 if there were any of the expenses and 0 if not. The amount of the expenses was not considered in the analysis. The term ‘prepared dishes' in Burkina Faso refers to dishes that are bought outside the home but consumed at home, mostly acquired in small restaurants directly settled on streets or from women preparing dishes at home and selling them to neighbours or on stalls in front of their homes.

The place of choice to purchase food was computed as 1 for market and 0 for any other response, which included street food sellers, small shops, house to house sellers and others.

### 2.5 Statistical analysis

Data entry, including quality checks, was performed with Epidata software, version 3.1. Data management was performed with SAS software version 8.0, and data analysis were performed using STATA software, version 15.0. The sampling design of the dataset was declared by defining enumeration areas as primary sampling units with the *svyset* STATA function. We used the *svy* STATA command to take into account the clusterization of the sample. As sample was self‐weighted no sampling weights were applied.

As the three surveys conducted were independent from each other (different samples selected each year) we were not able to analyse the data longitudinally, and thus we decided to pool the data of the three years in order to increase the statistical power of the analysis, and to test the potential impact of the year of data collection in the model by introducing interaction terms with the year of survey.

Also, we stratified the analysis by city (Ouagadougou versus Bobo‐Dioulasso) and by type of district within each city. The statistical office in Burkina Faso differentiates two type of districts in the urban settings, *loti* or structured (with amenities like access to pipe water and electricity, sewage, etc.) and *unloti* or unstructured (with no amenities due to rapid spontaneous urbanization). Thus, we present results for four strata: (i) Ouagadougou structured districts; (ii) Ouagadougou unstructured districts; (iii) Bobo‐Dioulasso structured districts; and (iv) Bobo‐Dioulasso unstructured districts.

**Figure 1:**
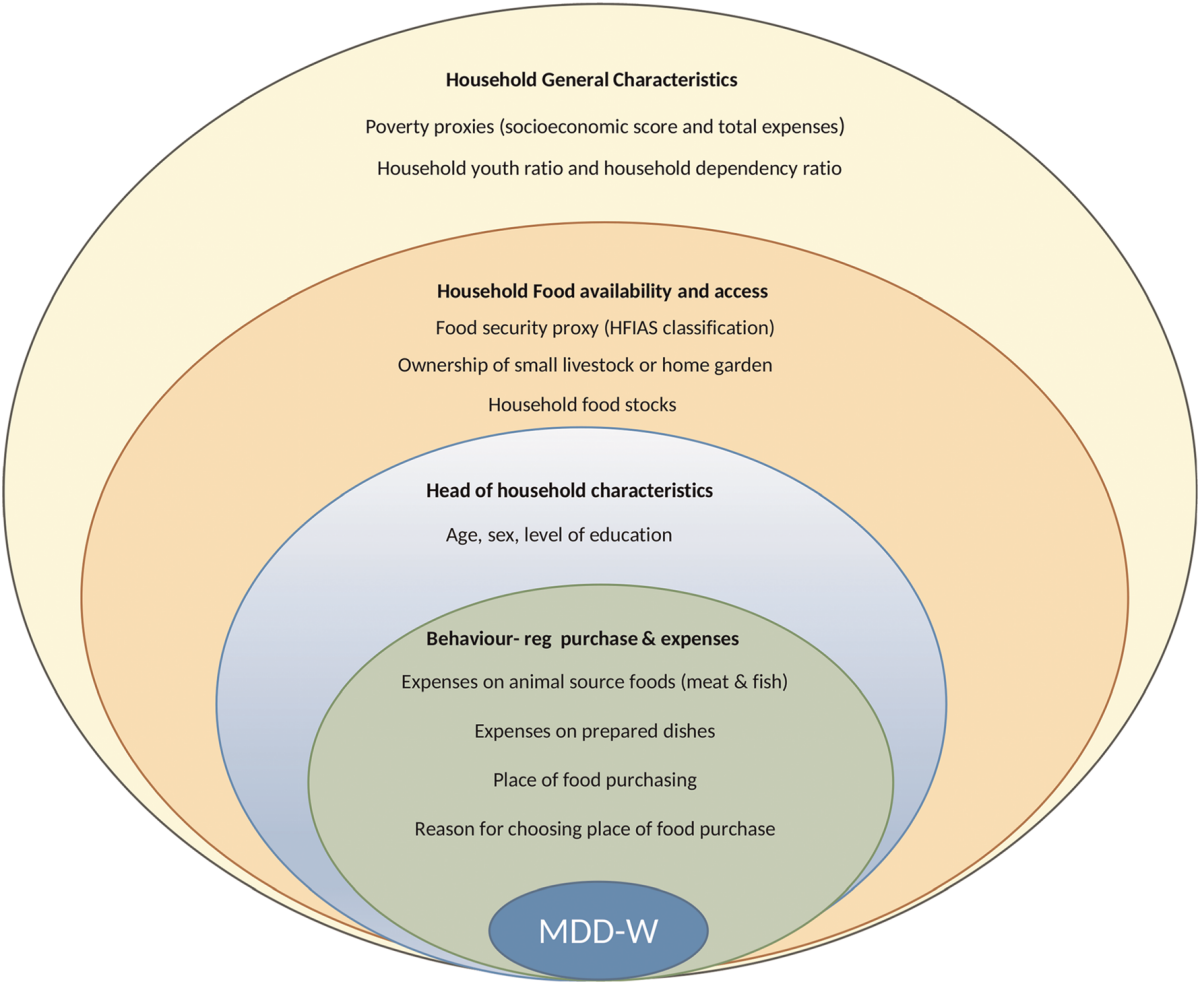
Conceptual framework for the assessment of MDD‐W associated factors

To assess the factors associated with MDD‐W we followed the conceptual framework depicted in Fig. 1.

In a first step, we carried out simple logistic regression analyses with MDD‐W as the dependent variable and each of the covariates included in the conceptual framework as the independent ones. Results of these bivariable analyses are included in Supplementary table 1. We then computed multivariable logistic regression models for each strata by using a manual forward stepwise procedure, entering all the covariates that were associated with MDD‐W at the *P*<0.10 level in the simple regression analyses, and following a distal‐proximal strategy as represented in the conceptual framework in Fig. 1.

In the multivariable analysis *P*‐values less than or equal to 0.05 were considered significant. The final multivariable models included all the variables that were significant in at least one of the four models (Ouagadougou‐*structured*, Ouagadougou‐*unstructured*, Bobo‐Dioulasso‐*structured* and Bobo‐Dioulasso‐*unstructured*).

Furthermore, in each of the strata we tested the interaction term of year of survey with each of the covariates, to assess if the association of MDD‐W with each of the covariates differed according to the year of survey. When the interaction term with one covariate and the year of survey was significant in at least in one strata, the interaction terms was kept in the four multivariable models and the estimates of those covariates were presented by year of survey.

Ethical considerations

The study design and the survey instrument were submitted for ethics review in accordance with Helsinki declaration, and were approved by the Ethics Committee of the Ministry of Health of Burkina Faso.

During the field work participants gave written informed consent before participating in the survey.

## 3 RESULTS

A total of 12 754 women were interviewed, 6 287 in Ouagadougou (64.5% living in structured districts and 35.5% in unstructured districts) and 6467 in Bobo‐Dioulasso (79.8% from structured and 20.2% from unstructured districts), see Table 1.

**Table 1:** Sample characteristics of Ouagadougou and Bobo Dioulasso stratified by type of district (2009‐2011)

		Ouagadougou		Bobo Dioulasso	
	Overall	Structured	Unstructured	Structured	Unstructured
	*N*=12754	*N*=4054	*N*=2233	*N*=5161	*N*=1306
		%	%	%	%
Total expenses (quintiles) 1st quintile	19.1	14.2	26.7	11.8	49.9
2nd quintile	20.7	17.4	28.2	19.4	23.8
3rd quintile	21.0	20.0	23.6	22.1	15.1
4th quintile	20.3	23.3	14.8	23.6	7.6
5th quintile	18.9	25.2	6.7	23.1	3.6
Socioeconomic score Low	33.7	22.1	60.6	19.4	80.8
Middle	30.3	31.6	29.6	33.4	15.4
High	34.0	34.0	9.9	47.2	3.8
Household dependency ratio score (mean)	4.2	3.6	3.6	4.8	4.6
Household youth ratio score (mean)	0.8	0.8	0.9	0.8	1.0
Age of head of household (mean)	41.2	41.5	37.5	42.7	40.6
Female headed households	10.5	10.9	7.2	12.0	8.5
Head of household education status None	46.2	39.9	50.6	44.3	65.8
Primary school	10.1	8.4	15.4	9.6	8.1
Secondary school	20.3	22.2	19.9	19.1	16.5
Higher education	23.5	29.5	14.1	27.0	6.7
Severely food insecure household	51.3	49.2	68.3	44.3	56.8
Household food stocks None	35.2	33.3	43.8	35.0	27.6
At least 5 kgs of cereals	35.1	33.1	41.4	35.6	36.5
At least 20 kgs of cereals	29.8	33.6	14.9	31.4	36.8
Household owns agriculture parcel	16.2	14.1	20.9	12.4	29.6
Household owns small livestock or poultry	18.4	16.8	14.8	14.8	44.3
Household reports montly expenses on meat	14.4	17.9	4.6	18.6	3.9
Household reports monthly expenses on fish	57.1	61.4	55.8	55.9	50.5
Household reports monthly expenses on prepared dishes	39.1	43.7	48.9	35.3	23.4
Place of choice to purchase food‐market	75.2	72.3	47.6	92.2	62.9
Reason to choose on place of food purchase‐large choice	16.5	28.2	14.4	15.5	12.6
Reason to choose on place of food purchase‐proximity	81.1	80.4	78.9	84.4	74.4

Among all urban women surveyed nearly 46% had not received any education and more than 50% lived in severely food insecure households, with more than 30% of households with no food stocks, although household and women's characteristics differed by city and type of district surveyed.

The structured districts showed better conditions than the unstructured districts in both cities. Overall, characteristics of the structured districts of Bobo‐Dioulasso and Ouagadougou were similar in terms of socioeconomic indicators. On the other hand, in Bobo‐Dioulasso the youth ratio, the proportion of female headed households, and the age of the head of the household were higher. On the other side Ouagadougou showed higher education levels for the head of the household although also a higher percentage of severely food insecure households. There were some differences in relation to purchasing behaviours, as fish and prepared dishes were more frequently reported by women in Ouagadougou than in Bobo‐Dioulasso. But in the latter food purchases were preferentially made on markets more often than in Ouagadougou.

The differences between the unstructured districts of the two cities were more marked. The unstructured districts of Bobo‐Dioulasso showed lower scores for the socioeconomic variables whereas households in Ouagadougou unstructured districts showed higher prevalence of severe food insecurity (68% as compared with 57%), lower food stocks and lower ownership of home gardens and small livestock (Table 1).

**Figure 2:**
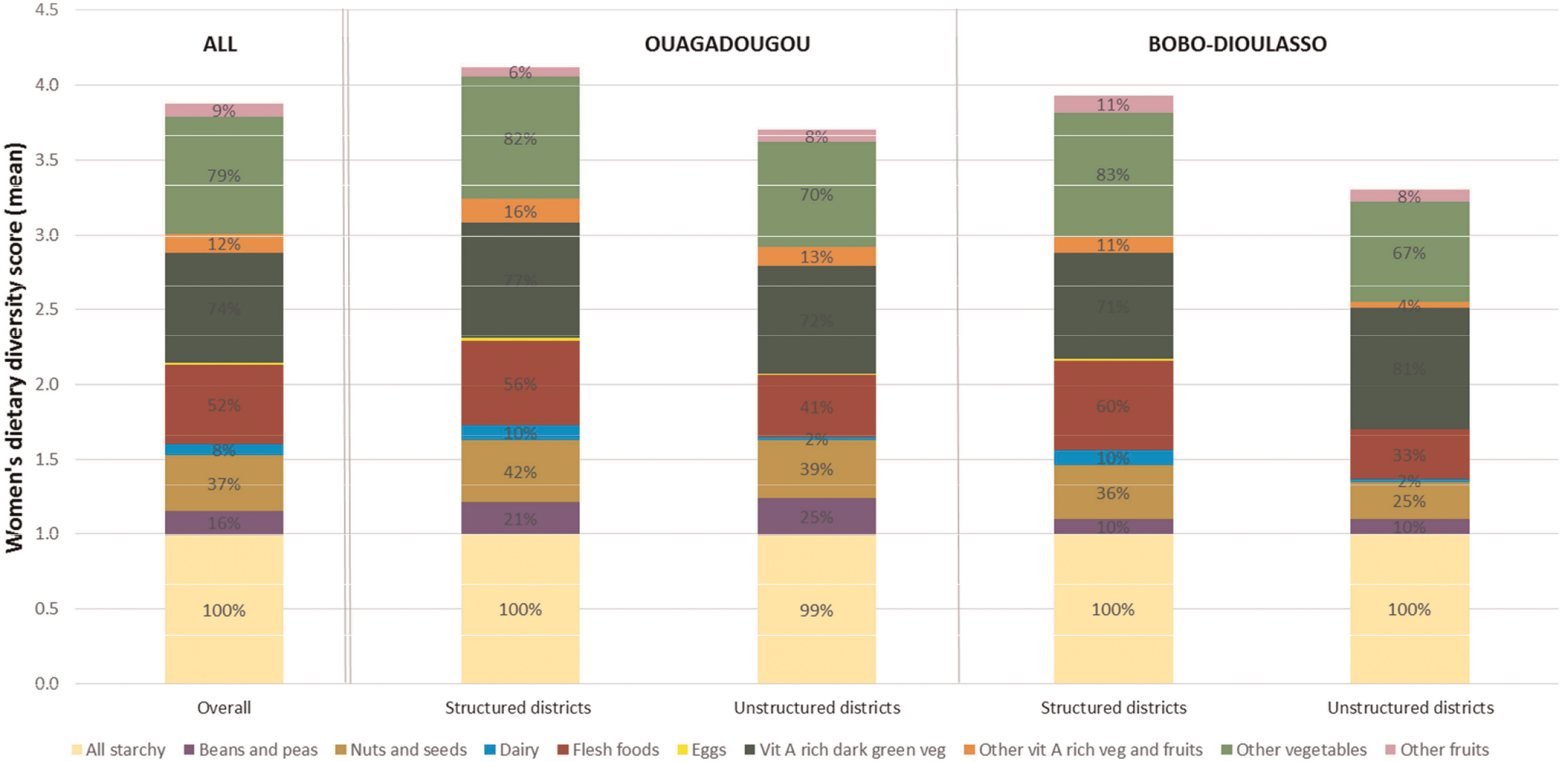
Mean WDDS‐10 and percentage of food groups consumed by women of reproductive age in Ouagadougou and Bobo‐Dioulasso for the 2009, 2010 and 2011 years period, by type of district

The mean dietary diversity of women of reproductive age for the overall sample was 3.8 food groups, and the differences were more pronounced between women living in different types of districts than between women living in different cities, the mean WDDS‐10 being 4.1 and 3.7 food groups for structured and unstructured districts in Ouagadougou, respectively, and 3.9 and 3.6 food groups for the structured and unstructured districts of Bobo‐Dioulasso, respectively (Fig. 2).

In the overall sample the most frequently reported food groups (FGs), apart from the all starchy group, were the DGLV followed by other vegetables, flesh foods and nuts and seeds. In Ouagadougou the nuts and seeds and the beans and pulses FGs were more frequently consumed than in Bobo‐Dioulasso. In both cities, the women living in unstructured districts reported consuming less frequently flesh foods, dairy products and nuts and seeds than their counterparts in the structured districts. Also, in Bobo‐Dioulasso the women living in unstructured districts consumed fruits and vegetables less frequently than those living in structured districts (except for the group of the DGLV).

For the sample comprising the whole period of analysis (years 2009, 2010 and 2011), the proportion of women reaching the MDD‐W was 30.6% overall, being 32.7% in the Ouagadougou subsample and 28.4% in Bobo‐Dioulasso, and showing lower estimates for the unstructured districts vs. the structured ones in both cities (24% vs. 38% in Ouagadougou and 13% vs. 32% in Bobo‐Dioulasso). The lowest prevalence of MDDW overall, and for each strata was recorded in year 2010.

In relation to the frequency of consumption of the various FGs in the overall sample, in 2009 there was a higher consumption of flesh foods, while in 2011 a higher consumption of the vegetables and fruits groups (Supplemental Figure 1).

Table 2 shows the results of the multivariable regression models for the overall sample and each of the four strata.

**Table 2:** Factors associated with MDD‐W in Ouagadougou and Bobo‐Dioulasso in the 2009‐2001 years period*

	Interaction term year of survey	Overall	Ouagadougou		Bobo‐Dioulasso	
		*N*=12277	Structured (*n*=3871)	Unstructured (*n*=2114)	Structured (*n*=5090)	Unstructured (*n*=1202)
Explanatory terms		AdjOR (CI 95%)	AdjOR (CI 95%)	Adj OR (CI 95%)	Adj OR (CI 95%)	Adj OR (CI 95%)
Year of survey		p=0.0028				
2009		Ref.	Ref.	Ref.	Ref.	Ref.
2010		0.7(0.5‐1.1)	1.1 (0.7‐2.0)	0.4 (0.1‐1.1)	0.8 (0.4‐1.3)	1.5(0.3‐6.2)
2011		1.0(0.7‐1.3)	1.4 (1.0‐2.2)	0.7(0.3‐1.6)	0.8(0.5‐1.4)	0.5(0.1‐1.9)
Total expenses (quintiles)						
1st quintile		Ref.	Ref.	Ref.	Ref.	Ref.
2nd quintile		1.5(1.2‐1.9)	1.6(1.1‐2.3)	1.2(0.8‐1.6)	1.7(1.2‐2.6)	1.6(1.0‐2.6)
3rd quintile		2.1(1.7‐2.6)	1.8(1.2‐2.7)	1.3(0.9‐1.9)	3.1(2.2‐4.5)	3.0(1.1‐7.7)
4th quintile		2.8(2.2‐3.4)	2.4(1.6‐3.7)	1.8(1.3‐2.6)	4.2(3.0‐6.0)	2.3(0.6‐8.4)
5th quintile		4.2(3.3‐5.4)	3.6(2.3‐5.7)	2.2(1.3‐3.5)	6.8(4.6‐10.1)	2.8(0.9‐8.5)
Socioeconomic score						
Low		Ref.	Ref.	Ref.	Ref.	Ref.
Middle		1.0(0.9‐1.2)	1.1(0.9‐1.4)	0.9(0.8‐1.1)	1.1(0.9‐1.4)	0.6(0.5‐5.2)
High		1.3(1.1‐1.6)	1.4(1.1‐2.1)	1.5(1.1‐2.3)	1.3(1.0‐1.7)	1.0(0.6‐1.7)
Household dependence ratio score						
Low		Ref.	Ref.	Ref.	Ref.	Ref.
Middle		0.9(0.8‐1.0)	0.9 (0.7‐1.2)	0.8(0.6‐1.1)	0.7(0.6‐0.9)	1.0(0.6‐1.7)
High		0.7(0.6‐0.8)	0.8(0.7‐1.0)	0.6(0.5‐0.8)	0.6(0.5‐0.7)	0.9(0.5‐1.5)
Age of head of household						
<30 years		Ref.	Ref.	Ref.	Ref.	Ref.
30‐39 years		0.9(0.8‐1.0)	0.8 (0.7‐1.0)	1.1(0.7‐1.6)	1.0(0.7‐1.2)	0.5(0.2‐0.8)
40‐49 years		0.7(0.6‐0.9)	0.7 (0.6‐0.9)	0.8(0.5‐1.2)	0.9(0.7‐1.2)	0.4(0.2‐0.8)
>50 years		0.4(0.3‐0.7)	0.6 (0.5‐0.8)	0.8(0.5‐1.2)	0.9(0.6‐1.2)	0.3(0.1‐1.0)
Household food security‐HFIAS						
Food secure		Ref.	Ref.	Ref.	Ref.	Ref.
Severely food insecure	2009	0.8(0.6‐0.9)	1.1(0.8‐1.6)	0.5(0.3‐0.7)	0.7(0.5‐1.0)	0.5(0.3‐1.1)
	2010	0.8(0.7‐0.9)	0.7(0.5‐0.9)	1.2(0.8‐1.9)	0.9(0.6‐1.2)	0.4(0.2‐0.6)
	2011	0.8(0.7‐0.9)	0.7(0.5‐0.9)	1.0(0.6‐1.5)	0.8(0.6‐1.1)	0.6(0.2‐1.6)
Household food stocks						
None		Ref.	Ref.	Ref.	Ref.	Ref.
At least 5 kgs of cereals	2009	0.8(0.7‐1.0)	1.0(0.7‐1.5)	0.8(0.4‐1.8)	0.9(0.6‐1.3)	0.4(0.1‐1.3)
	2010	1.1(0.9‐1.3)	1.0(0.7‐1.5)	1.5(0.8‐2.8)	1.0(0.7‐1.5)	0.6(0.2‐1.5)
	2011	1.1(0.9‐1.3)	1.0(0.7‐1.5)	1.8(1.2‐2.9)	0.9(0.6‐1.2)	1.8(0.8‐4.2)
At least 20 kgs of cereals	2009	0.9(0.8‐1.2)	1.0(0.7‐1.5)	1.0(0.5‐2.2)	0.9(0.6‐1.4)	0.9(0.3‐2.8)
	2010	1.1(0.9‐1.3)	1.2(0.7‐2.1)	4.8(2.3‐10.2)	0.8(0.5‐1.2)	0.8(0.4‐1.8)
	2011	1.0(0.8‐1.2)	0.9(0.6‐1.4)	1.6(0.7‐4.0)	0.9(0.6‐1.2)	1.8(0.6‐5.7)
Household owns small livestock						
No		Ref.	Ref.	Ref.	Ref.	Ref.
Yes	2009	0.9(0.7‐1.1)	1.0(0.6‐1.5)	0.7(0.3‐1.5)	0.7(0.5‐0.9)	1.9(0.9‐4.2)
	2010	1.0(0.9‐1.3)	0.9(0.7‐1.2)	0.8(0.3‐2.0)	1.3(0.9‐1.9)	1.1(0.4‐3.6)
	2011	1.0(0.8‐1.2)	0.9(0.7‐1.3)	0.8(0.5‐1.3)	1.1(0.8‐1.7)	1.8(1.0‐3.2)
Household owns home garden						
No		Ref.	Ref.	Ref.	Ref.	
Yes		1.1(0.9‐1.3	0.9 (0.7‐1.2)	1.3(0.7‐2.3)	1.2(0.9‐1.4)	1.7(1.0‐2.9)
HH reports montly expenses on meat						
No		Ref.	Ref.	Ref.	Ref.	Ref.
Yes		4.2(3.5‐5.1)	3.9(3.8‐5.4)	3.9(2.8‐5.5)	4.4(3.3‐5.9)	3.8(1.3‐11.1)
HH reports monthly expenses on fish						
No		Ref.	Ref.	Ref.	Ref.	Ref.
Yes		2.5(2.2‐2.9)	2.3(1.8‐2.9)	2.1(1.7‐2.8)	2.6(2.1‐3.2)	4.4(2.9‐6.8)
HH reports monthly expenses on prepared dishes						
No		Ref.	Ref.	Ref.	Ref.	Ref.
Yes		1.2(1.0‐1.3)	1.3(1.1‐1.6)	1.3(1.0‐1.6)	0.9(0.7‐1.1)	2.1(1.7‐2.7)
Place of choice to purchase food						
Other		Ref.	Ref.	Ref.	Ref.	Ref.
Market		1.1(1.0‐1.3)	1.4(1.1‐1.7)	0.9(0.7‐1.1)	1.7(1.1‐2.7)	2.0(1.0‐3.7)
Reason to choose place of food purchase‐large choice						
No		Ref.	Ref.	Ref.	Ref.	Ref.
Yes		1.4(1.2‐1.7)	1.5(1.2‐2.0)	1.4(0.9‐2.4)	1.3(1.0‐1.6)	2.0(1.4‐2.7)
Reason to choose place of food purchase‐proximity						
No		Ref.	Ref.	Ref.	Ref.	Ref.
Yes		1.0(0.9‐1.1)	1.0(0.8‐1.3)	0.7(0.6‐0.9)	1.1(0.9‐1.3)	1.4(0.8‐2.6)

* Multivariable models are run with registries that have no missing data for any of the covariates included, thus N does not match with initial number.

The likelihood of reaching MDD‐W increased significantly with higher total expenses in all four strata, whereas the relationship with higher socioeconomic score was significant only in Ouagadougou (although it was significant also in Bobo‐Dioulasso in the simple regression models, see Supplemental Table 1). The age of the head of the household was inversely related to MDD‐W: the older the head of the household, the less likely were the women to consume at least five FGs, the association being significant only in Ouagadougou‐*structured* and in Bobo‐Dioulasso‐*unstructured*.

Other sociodemographic variables showed significant association with MDD‐W in the simple models but lost significance when introduced in the multivariable model with the rest of covariates. In the simple models of all four strata the level of education of the head of the household, and the household receiving a regular income were positively associated with MDD‐W and the youth ratio inversely. In Bobo‐Dioulasso strata also the household's ownership of big livestock increased the likelihood of women reaching MDD‐W and household's reception of food aid decreased it. Finally, women living in Ouagadougou showed higher likelihood of reaching MDD‐W in 2011 as compared with year 2009, although the significance was lost in the multivariable models (See Supplemental Table 1).

The interaction terms of the year and selected food security variables were not significant for the overall sample but they were significant for selected strata as follows: (i) the HFIAS in Ouagadougou‐*structured* strata; (ii) the household's food stocks in Ouagadougou‐*unstructured*; and (iii) the owning of small livestock in Bobo‐Dioulasso‐*structured*. Thus, those interaction terms were kept in the multivariable model and the estimates for those covariates are reported by year of survey in each of the four strata (See Table 2).

In severely food insecure households the likelihood of reaching MDD‐W was lower overall, but the strength of this inverse association varied depending on the strata and the year.

Households with larger cereal stocks showed higher MDD‐W overall, although the association was significant only in the unstructured districts of Ouagadougou, and starker in year 2010.

The association between the owning of small livestock and MDD‐W showed different directions depending on the strata and year. In Ouagadougou the association seems to be inverse, that is, owning small livestock/poultry was associated with a lower likelihood of reaching MDD‐W, except in 2010 in Ouagadougou‐unstructured strata where the association observed was straightforward, although not significant.

On the other hand, in Bobo‐Dioulasso, owning small livestock was associated with a higher likelihood of reaching MDD‐W except for the structured districts in 2009, where the association is inverse and significant.

In households purchasing meat or fish in the previous month women were three to four times more likely to reach the MDD‐W, in the case of meat, and two to four times in the case of fish. Reporting monthly expenses on prepared dishes was associated with higher odds for women to reaching MDD‐W, between 1.3 to 2.1 times depending on the strata.

Purchasing foods on the market also showed a consistent and positive association with a higher dietary diversity of women, as well as choosing the place of purchase based on a larger choice, compared with choosing the place because of its proximity, which was inversely related to MDD‐W in the unstructured districts of Ouagadougou.

## 4 DISCUSSION

Overall 31% of women living in urban Burkina Faso consumed at least five food groups out of the ten predefined food groups to measure MDD‐W (33% of WRA in Ouagadougou and 28% in Bobo‐Dioulasso). In both cities the prevalence of MDD‐W was significantly lower in the unstructured districts as compared with the structured ones.

There is paucity of data from other urban settings to compare our results with, but in surveys conducted by the German development cooperation agency (GIZ) in 2015‐2016 in rural settings of ten different countries, MDD‐W ranged from 7% in Ethiopia to 57% in Zambia (Kennedy, Keding, Evang, Nodari, & Scheerer, 2017). In another rural survey conducted in Tanzania during the lean season MDD‐W was recorded at 46% among women 15 to 35 years of age (Ochieng, Afari‐Sefa, Lukumay, & Dubois, 2017). In the South West region of Burkina Faso, MDD‐W prevalence was 38% in a season of medium food availability (Gina Kennedy et al., 2017), so not too different from what we observed in the structured districts of Ouagadougou but, surprisingly, 10 percentage points higher than in Bobo‐Dioulasso. In two rural settings of Burkina Faso MDD‐W post‐harvest estimations (49% and 30%) were reported to be higher than the lean season estimates (26% and 23% respectively) within the same populations suggesting that seasonality has an impact on the indicator, at least in rural communities of this context (Custodio, Kayitakire, & Thomas, 2015).

The fact that Ouagadougou showed higher MDD‐W prevalence overall may be related to its condition of the capital city and thus markets may offer a wider range of food products. Furthermore, data was collected during the post‐harvest season in Bobo‐Dioulasso and in the beginning of the lean season in Ouagadougou, suggesting that the differences observed could have been starker if the data had been collected at the same time of the year in both cities. Indeed, in Ouagadougou a lower dietary diversity during the lean season was already reported by another study (Becquey et al., 2012).

The overall low values of dietary diversity in our sample may be related to the effects of the food price crises which started in 2008 (Brinkman, de Pee, Sanogo, Subran, & Bloem, 2010), as this type of crisis may especially affect urban households, as they depend on the market for food provisioning, and see their purchasing power reduced. The impact of the food price crisis on the dietary diversity of the population of Ouagadougou's was already reported for 2008 (Martin‐Prevel et al., 2012), and our results may be showing the remaining effects of it. Also, there were fluctuations in MDD‐W depending on the year of study, which were more marked in Ouagadougou than in Bobo‐Dioulasso. Both cities showed lower MDD‐W estimations for the year 2010. In that year, there was a food security crisis in the Sahel due to poor rainfall, which may have caused the low dietary diversity recorded in June‐July 2010. Furthermore, the stark deterioration of dietary diversity in Ouagadougou in 2010 has been previously associated with the floods that occurred in Burkina Faso in September 2009. More than 44% of the Ouagadougou sample households were affected, and showed a dietary diversity significantly lower than the unaffected ones (Vernay M, 2012).

Apart from the 'all starchy' food group, the vegetables FGs were the most frequently consumed by women living in urban Burkina Faso, the 'other vegetables' FG being more frequently consumed in structured districts and the DGLV food group in the unstructured ones. As shown in other studies, the DGLV are frequently consumed in West and Southern Africa. The 'eggs', 'vitamin A‐rich fruits and vegetables' and 'other fruits' FGS were rarely reported in our study population, which is consistent with other MDD‐W surveys conducted in Africa and Asia, with the exception of Tajikistan, Togo and Cambodia where eggs consumption is not that rare (Kennedy et al., 2017) (F. Nicolò, Nowak, Mak, & Lee, 2015). The higher consumption of Vitamin A‐rich vegetables and fruits in Ouagadougou compared with Bobo‐Dioulasso may be related to the different time of the year of data collection, as the mango season was over at the time the surveys were carried out in Bobo‐Dioulasso.

The dairy were consumed by 8% of the women overall, which increased to 10% in the structured districts of the two cities and by 2% of the women in the unstructured ones, with no difference between the cities. In the majority of other MDD‐W studies the dairy consumption reported was low, with high dairy consumption only related to specific cultural traditions or interventions targeting to improve availability of safe and affordable milk (Kennedy et al., 2017). Around 50% of women consumed flesh foods overall, although in both cities flesh foods were also consumed more often in the structured districts (~60%) versus the unstructured districts (~30‐40%). The consumption of animal source foods has been positively related to the level of endowment of the communities in other African countries (Herrador et al., 2015), and in Burkina Faso, the prices of animal‐source foods were described as prohibitive for urban poor (13) who are more numerous in the unstructured districts.

The total expenses of the household was indeed one of the factors strongly related to MDD‐W among urban women in Burkina Faso, with women from households in the highest quintile of total expenses being up to four times more likely to reach MDD‐W than their counterparts living in households in the lowest quintile, and this association remained in all four strata. The socioeconomic score showed a much weaker association and not significant across all strata, suggesting that short term income proxies have a higher impact on MDD‐W than longer term income proxies like quality of housing, owning of assets or facilities. In another study among urban women in Burkina Faso no association was found either between the socioeconomic index and their dietary diversity index (Savy et al., 2008). Furthermore, in studies conducted in rural settings findings are similar, with a study in rural settings of Burkina Faso showing no association between socioeconomic scores and MDD‐W (Custodio E et al., 2015), and the association being significant only in few of the selected rural areas in the countries studied by GIZ (Kennedy et al., 2017). In fact, the associations between dietary diversity scores and socioeconomic scores described in the literature so far were mainly observed with the household dietary diversity score, that represents access to a variety of foods rather than food consumption (Hatløy, Hallund, Diarra, & Oshaug, 2000) (Hoddinott J & Yohannes Y, 2002). The discrepancies between the household dietary scores and the individual dietary scores such as MDD‐W may be related to the intra‐household allocation of foods.

Nevertheless, households reporting monthly expenses on meat or fish showed a higher likelihood of reaching MDD‐W in this urban sample. Other studies have shown that the monthly food expenditures influence dietary diversity, suggesting that households that purchased foods at markets consistently have higher dietary diversity (also showing a high correlation with animal‐source foods) in Subsaharan Africa (Rajendran et al., 2017). The positive association found between expenses on prepared dishes and MDD‐W may be related to the importance of buying ready‐to‐eat foods and snacking among urban food practices of Burkina Faso (Becquey & Martin‐Prevel, 2010). Although traditionally the purchase of non‐home prepared foods has been associated with low income households and low‐density diets, studies in other African urban settings have identified that the adequacy of energy and nutrients intake does not differ between consumers of non‐home prepared foods and non‐consumers (van't Riet, den Hartog, & van Staveren, 2002).

In this study, the association of MDD‐W with food security variables varied according to the year, the strata and the food security variable analysed. Women living in severely food insecure households as measured by the HFIAS indicator were less likely to reach MDD‐W for the population overall, although this was more marked in years 2009 and 2010. The level of cereal stocks in the household also showed a positive association with MDD‐W for the overall sample and in all years and strata, although it was only significant in the unstructured districts of Ouagadougou (which is the strata showing the lowest levels of food stocks in general) and for the year 2010, the year with the worst living conditions in Ouagadougou out of the three studied. Our consideration is that households with cereals stocks will be in a position to purchase non starchy food products in the market, thus contributing to a more diverse diet.

Although home gardens were not positively associated with women's dietary diversity in the overall sample or in the structured districts, they were associated with MDD‐W in the unstructured districts of both cities, and significantly in Bobo‐Dioulasso. This is in line what was found in a study conducted in Tanzania (Keding, Msuya, Maass, & Krawinkel, 2012), as well as in Cambodia, Malawi, India and Kenya within the GIZ study (Gina Kennedy (first) et al., 2017).

With household owning of small livestock and poultry there was a similar effect, with no association among the pooled sample of urban women in Burkina Faso, but showing a positive and consistent association with MDD‐W in Bobo‐Dioulasso unstructured districts. In other strata the direction of the association varied depending on the year. Thus, for the year 2010 owning small livestock showed a protective effect in Ouagadougou unstructured districts whereas in hear 2009 and 2011 the association was reversed, suggesting that in times of hardship owning livestock was protective whereas in other times it may be a sign of poverty. However, as other studies point out, providing home gardens or small livestock may not have an impact on MDD‐W if these actions are not accompanied by women empowerment and nutrition knowledge interventions (McDermott, Aït‐Aïssa, Morel, & Rapando, 2013).

The behavioural variables included in this study offer interesting insights, such as the positive association between the fact that household chose to buy foods at markets as opposed to street vendors or small shops and MDD‐W, which was independent from household expenses or other socioeconomic variables. This may be related to the exposure to a higher food variety, as this is also captured by the positive association between choosing the place to purchase due to large food choice and MDD‐W and the inverse association with choosing the place of purchase due to proximity. The role of markets in the household's diet quality had already been described in Malawi (Koppmair, Kassie, & Qaim, 2017), and in relation to individuals dietary diversity, it has been suggested that knowledge interventions may have impact on the dietary diversity of children in areas with relatively good market access, whereas no impact was observed in remote areas with limited access to the market (Hirvonen K, Hoddinott J, Minten B, & Stifel D, 2016).

### 4.1 Strengths and limitations

The present results are not representative of the entire country as they are samples of two cities, Ouagadougou and Bobo‐Dioulasso. Also, the cross sectional nature of the study limited the interpretation of the changes in MDD‐W observed across the study period.

However, having data collected in three different years, on reasonably large sample sizes, allowed us to test for interactions and explore the impact of the year of survey on the MDD‐W and associated factors. Also, strength of the survey was the careful design and implementation of the dietary recall methodology due to the extended experience of the investigators with previous studies in Burkina Faso, as well as the use of the 24 hour open recall which is considered best practice for measuring MDD‐W.

## 5 CONCLUSIONS

The prevalence of MDD‐W in urban Burkina Faso is around 30%, being significantly higher among women living in structured districts as compared with women from unstructured districts. The food groups most frequently reported apart from the all starchy were the dark green vegetables, other vegetables, flesh foods and nuts and seeds.

Of the three years studied 2010 registered the lowest values for MDD‐W. Total expenses and animal source food expenses were highly associated with the MDD‐W as well as food insecurity.Ownership of small livestock and home gardens showed direct and inverse association depending on the setting and year of study. Purchasing foods in markets and choosing the place of purchase in relation to the availability of choice and not to proximity showed a positive association with the MDD‐W.

Dietary diversity interventions should be targeted to households with low expenses, the purchase in markets to be exposed to larger diversity should be encouraged as a means to increase the diversity of women's diets.

## CONFLICTS OF INTEREST

The authors declare that they have no conflicts of interest.

## CONTRIBUTIONS

YMP designed and led the research project. BN and YK conducted the research. EC FK and YMP designed the research analysis of the study. EC, SF, TK and AT performed data analysis and interpretation. EC wrote the paper. All authors revised the manuscript critically for important intellectual contributions and approved the final version.

## Supporting information

Figure S1. Women's food groups consumption in urban Burkina Faso by year of survey.
Table S1. Factors associated with MDDw in Ouagadougou and Bobo Dioulasso. Household socioeconomic variables. Results from bivariate logistic regressions*
