# Supplementary material for: Minimum dietary diversity among women of reproductive age in urban Burkina Faso
Source: Matern Child Nutr. 2019 Dec 19;16(2):e12897. doi: 10.1111/mcn.12897 (PMC7083435; doi:10.1111/mcn.12897)
Supplement: Supplementary file 1 — Figure S1. Women's food groups consumption in urban Burkina Faso by year of survey. Table S1. Factors associated with MDDw in Ouagadougou and Bobo Dioulasso. Household socioeconomic variables. Results from bivariate logistic regressions* [file MCN-16-e12897-s001.pdf]

Supplementary Appendix

Supplemental Figure 1: Women's food groups consumption in urban Burkina Faso by year of survey

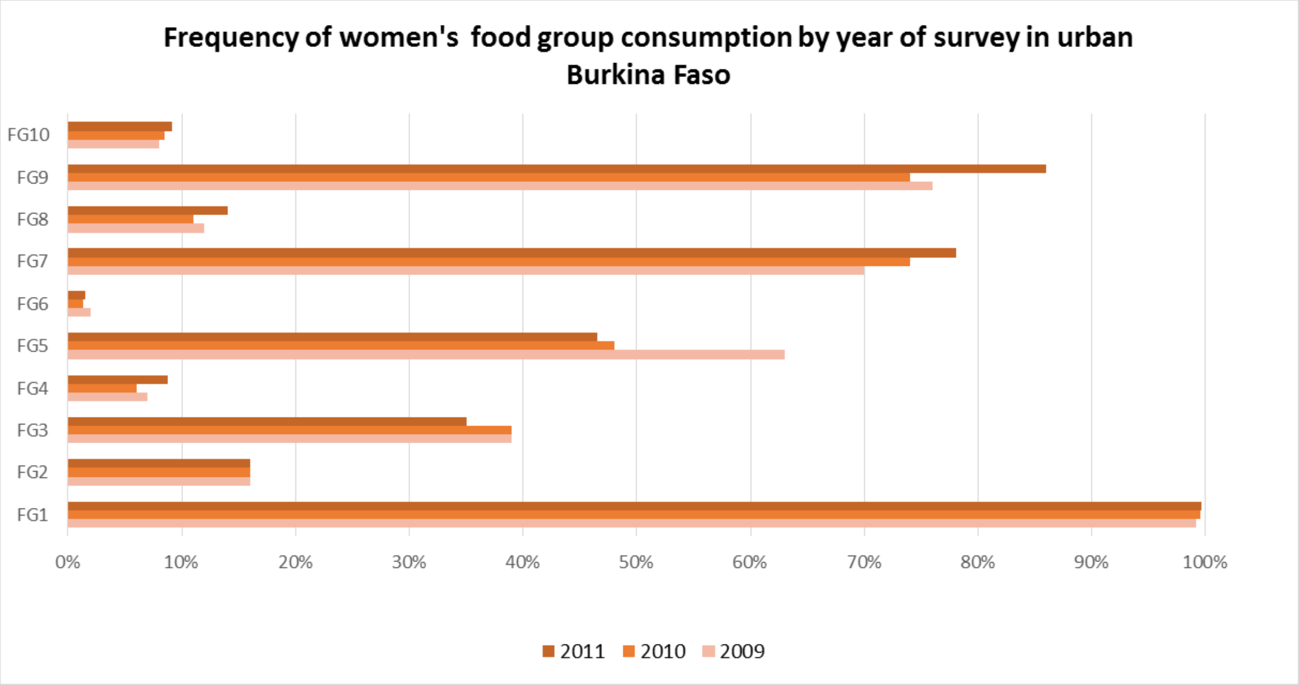

FG1: All starchy **FG2:** Beans and peas **FG3:** Nuts and seeds **FG4**:Dairy **FG5**:Flesh foods **FG6:**Eggs **FG7**:Vit A rich dark green vegetables **FG8:**Other vit A rich vegetables and fruits **FG9:** Other vegetables **FG10:** Other fruits

Supplemental Table 1

Table S1: Factors associated with MDDw in Ouagadougou and Bobo Dioulasso. Household socioeconomic variables. Results from bivariate logistic regressions*															
	Overall			Ouagadougou						Bobo Dioulasso					
	N=12754			Structured (n=4054)			Unstructured (n=2233)			Structured (n=5161)			Unstructured (n=1306)		
**COVARIATES**	n	MDDw %	OR (CI 95%)	n	MDDw %	OR (CI 95%)	n	MDDw %	OR (CI 95%)	n	MDDw %	OR (CI 95%)	n	MDDw %	OR (CI 95%)
**Year of survey**															
2009	4043	32.0	Ref.	1385	38.9	Ref.	670	22.8	Ref.	1527	34.1	Ref.	413	14.5	Ref.
2010	4144	27.3	0.8(0.7-0.9)	1232	32.1	0.7(0.6-0.9)	743	20.7	0.9(0.7-1.1)	1732	31.0	0.9(0.8-1.0)	462	11.3	0.7(0.5-1.1)
2011	4515	32.2	1.0(0.9-1.1)	1437	40.8	1.1(0.9-1.3)	806	28.0	1.3(1.0-1.7)	1831	31.4	0.9(0.8-1.0)	431	14.4	1.0(0.7-1.5)
p			<0.001			<0.001			0.003			0.117			0.258
**Total expenses (quintiles)**															
1st quintile	2411	11.6	Ref.	566	14.0	Ref.	588	16.7	Ref.	610	8.4	Ref.	647	7.9	Ref.
2nd quintile	2317	18.3	1.7(1.5-2.0)	691	23.6	1.9(1.4-2.6)	622	19.5	1.3(1.0-1.6)	995	15.5	2.0 (1.4-2.8)	309	13.6	1.8(1.2-2.8)
3rd quintile	2645	27.4	2.9(2.5-3.4)	798	30.0	2.6(2.0-3.5)	518	25.3	1.7(1.3-2.3)	1133	27.9	4.2 (3.1-5.8)	196	19.9	3.0(1.8-4.6)
4th quintile	2563	38.9	4.9(4.2-5.6)	926	42.8	4.6(3.5-6.0)	325	34.8	2.7(1.9-3.7)	1213	38.7	6.9(5.1-9.4)	99	20.2	3.0(1.7-5.2)
5th quintile	2378	57.3	10.2(8.8-11.9)	997	61.6	9.9(7.5-12.9)	149	44.3	4.0(2.7-5.9)	1185	55.9	13.9(10.2-18.9)	47	42.6	8.7(4.5-16.5)
p			<0.001			<0.001			<0.001			<0.001			<0.001
**Socioeconomic score**															
Low	4293	16.7	Ref.	890	17.9	Ref.	1347	19.8	Ref.	1001	16.3	Ref.	1055	11.9	Ref.
Middle	3845	25.0	1.7(1.5-1.9)	1268	27.7	1.8(1.4-2.2)	653	24.7	1.3(1.1-1.7)	1723	24.8	1.7 (1.4-2.1)	201	11.9	1.0(0.6-1.6)
High	4564	48.3	4.7(4.2-5.2)	1858	53.7	5.3(4.4-6.5)	219	48.0	3.7(2.8-5.0)	2437	44.2	4.1 (3.8-4.9)	48	48.0	6.8(3.8-12.2)
p			<0.001			<0.001			0.03			<0.001			<0.001
**Household dependency ratio score**															
Low	3876	33.9	Ref.	801	43.1	Ref.	425	30.6	Ref.	2141	36	Ref.	509	13.8	Ref.
Middle	4759	30.3	0.8(0.8-0.9)	1603	39.3	0.9(0.7-1.0)	777	25.3	0.8(0.6-1.1)	1864	29.3	0.7 (0.6-0.8)	515	13.4	1.0(0.7-1.4)
High	3917	27.7	0.7(0.7-0.8)	1546	33.4	0.7(0.6-0.8)	965	20.1	0.6(0.5-0.7)	1130	29.9	08 (0.7-0.9)	276	12.7	0.9(0.6-1.4)
p			<0.001			<0.001			0.008			<0.001			0.947
**Household youth ratio score**															
Low	3776	36.1	Ref.	1003	45.0	Ref.	408	30.2	Ref.	2002	36	Ref.	363	17.6	Ref.
Middle	4746	31.3	0.8(0.7-0.9)	1506	40.7	0.8(0.7-1.0)	730	24.5	0.8(0.6-1.0)	1998	31.6	0.8 (0.7-0.9)	512	12.7	0.7(0.5-1.0)
High	4162	24.7	0.6(0.5-0.6)	1499	29.8	0.5(0.4-0.6)	1077	21.5	0.6(0.5-0.8)	1155	26.4	0.6 (0.5-0.7)	431	10.4	0.5(0.4-0.8)
p			<0.001			<0.001			0.01			<0.001			0.009
**Sex head of household**															
Female	1327	30.4	Ref.	437	37.7	Ref.	159	18.9	Ref.	620	32.2	Ref.	111	13.6	Ref.
Male	11374	30.6	1.0(0.9-1.1)	3579	36.6	1.0(0.8-1.2)	2059	24.4	1.4(0.9-2.1)	4541	32.6	1.0(0.8-1.2)	1195	10.8	1.3(0.7-2.4)
p			0.93			0.668			0.105			0.856			0.403
**Age of head of household**															
<30 years	1320	31.1	Ref.	379	39.3	Ref.	367	26..2	Ref.	445	31.5	Ref.	129	19.4	Ref.
30-39 years	4699	31.4	1.0(0.9-1.2)	1463	39.3	1.0(0.8-1.3)	1020	26	1.0(0.8-1.3)	1727	32.8	1.1(0.9-1.3)	489	13.5	0.6(0.4-1.1)
40-49 years	3943	29.9	0.9(0.8-1.1)	1283	36.8	0.9(0.7-1.1)	585	20.5	0.7(0.5-1.0)	1660	32.1	1.0(0.8-1.3)	415	12.8	0.6(0.4-1.0)
>50 years	2644	30.3	1.0(0.8-1.1)	881	35	0.8(0.6-1.1)	242	20.3	0.7(0.5-1.1)	837	32.4	1.0(0.8-1.3)	198	10.6	0.5(0.3-0.9)
p			0.477			0.153			0.062			0.937			0.162
**Head of household education status**															
None	5871	23.4	Ref.	1604	30.3	Ref.	1125	19.9	Ref.	2284	25.0	Ref.	858	10.6	Ref.
Primary school	1276	31.0	1.5(1.3-1.7)	337	30.9	1.0(0.8-1.3)	340	25.3	1.4(1.0-1.8)	494	37.3	1.8(1.5-2.2)	105	21.0	2.2(1.3-3.7)
Secondary school	2573	29.2	1.4(1.2-1.5)	892	33.6	1.2(1.0-1.4)	440	26.1	1.4(1.1-1.8)	986	29.9	1.3(1.1-1.5)	255	16.5	1.7(1.0-2.5)
Higher education	2975	45.7	2.8(2.5-3.0)	1180	52.3	2.5(2.2-2.9)	312	34.6	2.1(1.6-2.8)	1396	44.1	2.4(2.1-2.7)	87	21.8	2.4(1.4-4.1)
p			<0.001			<0.001			<0.001			<0.001			<0.001
**Head of household receives regular aid (bourse/aid)**															
No	12084	31.0	Ref.	3885	37.8	Ref.	2115	24.2	Ref.	4857	32.9	Ref.	1227	13.9	Ref.
Yes	613	21.2	0.6(0.5-0.7)	131	29.8	0.7(0.5-1.0)	103	21.4	0.9(0.5-1.4)	302	21.5	0.6 (0.4-0.7)	77	5.2	0.3(0.1-0.9)
p			<0.001			0.054			0.786			<0.001			0.016
**Head of household has regular income**															
Yes	2851	42.5	Ref.	2840	32.8	Ref.	367	27.8	Ref.	1195	42.2	Ref.	1191	12.2	Ref.
No	9846	27.1	2.0(1.8-2.2)	1176	48.1	2.0(1.7-2.3)	1851	23.3	1.3(1.0-1.6)	3964	29.3	1.8(1.5-2.0)	113	25.7	2.5(1.6-3.9)
p			<0.001			<0.001			0.048			<0.001			<0.001
**Household food security-HFIAS categorization**															
Food secure + MM Food Secure	6182	40.6	Ref.	2039	49.7	Ref.	705	32.8	Ref.	2874	40.3	Ref.	564	19.2	Ref.
Severely food insecure	6519	21.0	0.4(0.3-0.4)	1977	25	0.3(0.3-0.4)	1514	20	0.5(0.4-0.6)	2286	22.1	0.4(0.4-0.5)	742	8.9	0.4(0.3-0.6)
p			<0.001			<0.001			<0.001			<0.001			<0.001
**Household food stocks**															
None	4465	22.0	Ref.	1336	25.2	Ref.	973	16.9	Ref.	1807	24.7	Ref.	349	9.2	Ref.
At least 5 kgs of cereals	4452	28.7	1.4(1.3-1.6)	1328	33.6	1.1(0.9-1.4)	915	26.9	1.8(1.5-2.3)	1733	30.5	1.3 (1.2-1.5)	476	12.0	1.3(0.9-2.1)
At least 20 kgs of cereals	3784	42.8	2.7(2.4-2.9)	1352	53.6	3.4(2.9-4.0)	331	37.2	2.9(2.2-3.9)	1620	42.5	2.2(1.9-2.6)	481	17.7	2.1(1.4-3.3)
p			<0.001			<0.001			<0.001			<0.001			0.001
**Household owns vegetable garden**															
No	10649	32.0	Ref.	3452	40.1	Ref.	1757	24	Ref.	4520	32.9	Ref.	920	12.2	Ref.
Yes	2053	23.1	0.7(0.6-0.7)	564	22	0.4(0.3-0.5)	462	24.2	1.0(0.8-1.3)	641	27.5	0.8 (0.6-0.9)	386	16.1	1.4(1.0-1.9)
p						<0.001			0.899			0.005			0.033
**Household owns ox/bullock**															
No	12466	30.5	Ref.	3988	37.6	Ref.	2206	24.1	Ref.	5079	32.0	Ref.	1193	12.4	Ref.
Yes	236	33.1	1.1(0.9-1.5)	28	35.7	0.9(0.4-2.0)	13	15.4	0.6(0.1-2.6)	82	48.8	2.0 (1.3-3.1)	113	23.0	2.1(1.4-3.4)
p						0.84			0.443			0.002			0.003
**Household owns small livestock and/or poultry**															
No	10355	31.1	Ref.	3339	38.4	Ref.	1890	24.3	Ref.	4398	31.7	Ref.	728	10.7	Ref.
Yes	655	28.3	0.9(0.8-1.0)	677	33.4	0.8(0.7-1.0)	329	22.5	0.9(0.7-1.2)	763	35.3	1.2 (1.0-1.4)	578	16.6	1.7(1.2-2.3)
p			<0.001			0.013			0.48			0.057			<0.001
**Household reports montly expenses on meat**															
No	10868	24.8	Ref.	3297	30.4	Ref.	2116	22.4	Ref.	4200	25.3	Ref.	1255	12.0	Ref.
Yes	1834	64.9	5.6(5.0-6.2)	719	70.4	5.4(4.6-6.5)	103	57.3	4.6(3.1-7.0)	961	62.6	4.9(4.3-5.7)	51	45.1	6.0(3.4-10.7)
p			<0.001			<0.001			<0.001			<0.001			<0.001
**Household reports monthly expenses on fish**															
No	5456	23.8	Ref.	1550	31.1	Ref.	985	17.9	Ref.	2275	26.4	Ref.	646	6.4	Ref.
Yes	7246	35.6	1.8(1.6-1.9)	2466	41.6	1.6(1.4-1.8)	1234	29.0	1.9(1.5-2.3)	2886	36.9	1.6(1.4-1.8)	660	20.2	3.7(2.6-5.4)
p			<0.001			<0.001			<0.001			<0.001			<0.001
**Household reports monthly expenses on prepared dishes**															
No	7736	31.5	Ref.	2260	39.7	Ref.	1135	24.4	Ref.	3340	34.3	Ref.	1001	11.3	Ref.
yes	4966	29.1	0.9(0.8-1.0)	1756	34.8	0.8(0.7-0.9)	1084	23.6	1.0(0.8-1.2)	1821	28.5	0.8(0.7-0.9)	305	20.0	2.0(1.4-2.8)
p			0.006			0.001			0.732			<0.001			<0.001
**Place of choice to purchase food**															
Other	3144	17.8	Ref.	1110	20.8	Ref.	1150	21.2	Ref.	4749	33.9	Ref.	481	7.3	Ref.
Market	9480	34.7	2.4(2.2-2.7)	2876	44	3.0(2.5-3.5)	1041	26.6	1.3(1.1-1.6)	403	12.7	0.3(0.2-0.4)	814	17.1	2.6(1.6-3.5)
p			<0.001			<0.001			0.005			<0.001			<0.001
**Reason to choice place of food purchase-large choice**															
No	10538	27.7	Ref.	3176	33.4	Ref.	1875	21.4	Ref.	4355	30.4	Ref.	1132	11.9	Ref.
Yes	2086	44.6	2.1(1.9-2.3)	810	53.8	2.3(2.0-2.7)	316	37.7	2.2(1.7-2.9)	797	42.3	1.7(1.4-2.0)	163	23.9	2.3(1.6-3.5)
p			<0.001			<0.001			<0.001			<0.001			<0.001
**Reason to choice place of food purchase-proximity**															
No	2382	32.8	Ref.	783	41.9	Ref.	462	30.7	Ref.	805	33.9	Ref.	332	11.8	Ref.
Yes	10242	30.0	0.9(0.8-1.0)	3203	36.5	0.8(0.7-0.9)	1729	21.9	0.6(0.5-0.8)	4347	31.9	0.9(0.8-1.1)	963	14.0	1.2(0.8-1.8)
p			0.0067			0.005			<0.001			0.265			0.392

*Totals may not add to N due to missings in specific variables
